# High-Frequency Exon Deletion of DNA Cross-Link Repair 1C Accounting for Severe Combined Immunodeficiency May Be Missed by Whole-Exome Sequencing

**DOI:** 10.3389/fgene.2021.677748

**Published:** 2021-08-04

**Authors:** Feifan Xiao, Yulan Lu, Bingbing Wu, Bo Liu, Gang Li, Ping Zhang, Qinhua Zhou, Jinqiao Sun, Huijun Wang, Wenhao Zhou

**Affiliations:** ^1^Center for Molecular Medicine, Children’s Hospital of Fudan University, National Children’s Medical Center, Shanghai, China; ^2^Department of Immunology, Children’s Hospital of Fudan University, National Children’s Medical Center, Shanghai, China; ^3^Key Laboratory of Neonatal Diseases, Ministry of Health, Department of Neonates, Children’s Hospital of Fudan University, National Children’s Medical Center, Shanghai, China

**Keywords:** severe combined immunodeficiency, *DCLRE1C* gene, copy number variation, single nucleotide variation, sequencing

## Abstract

Next-generation sequencing (NGS) has been used to detect severe combined immunodeficiency (SCID) in patients, and some patients with DNA cross-link repair 1C (*DCLRE1C*) variants have been identified. Moreover, some compound variants, such as copy number variants (CNV) and single nucleotide variants (SNV), have been reported. The purpose of this study was to expand the genetic data related to patients with SCID carrying the compound *DCLRE1C* variant. Whole-exome sequencing (WES) was performed for genetic analysis, and variants were verified by performing Sanger sequencing or quantitative PCR. Moreover, we searched PubMed and summarized the data of the reported variants. Four SCID patients with *DCLRE1C* variants were identified in this study. WES revealed a homozygous deletion in the *DCLRE1C* gene from exons 1–5 in patient 1, exons 1–3 deletion and a novel rare variant (c.92T>C, p.L31P) in patient 2, exons 1–3 deletion and a novel rare variant (c.328C>G, p.L110V) in patient 3, and exons 1–4 deletion and a novel frameshift variant (c.449dup, p.His151Alafs*20) in patient 4. Based on literature review, exons 1–3 was recognized as a hotspot region for deletion variation. Moreover, we found that compound variations (CNV + SNV) accounted for approximately 7% variations in all variants. When patients are screened for T-cell receptor excision circles (TRECs), NGS can be used to expand genetic testing. Deletion of the *DCLRE1C* gene should not be ignored when a variant has been found in patients with SCID.

## Introduction

Severe combined immunodeficiency (SCID), one of the most severe forms of primary immunodeficiency diseases, is characterized by a deficiency of T-cell, B-cell, and sometimes NK-cell responses to infections. The reported incidence of SCID ranges from one per 50,000 to one per 1,00,000 live births (Lipstein et al., 2010). The affected patients fail to clear infections and usually die early in life, even with treatment. Studies have shown that genetic abnormalities are associated with the development of SCID. To date, more than 40 genes have been reported to be associated with SCID.

The DNA cross-link repair 1C (*DCLRE1C*) gene, also known as *ARTEMIS*, is located on chromosome 10p13. It encodes the nuclease ARTEMIS, a protein with 5ꞌ–3ꞌ exonuclease activity for single-stranded DNA. ARTEMIS plays an important role in V(D)J recombination that occurs during B- and T-cell development. Variations in the *DCLRE1C* gene is associated with autosomal recessive SCID by affecting the V(D)J recombination. In 2001, Moshous et al. (2001) reported 13 patients with SCID who carried *DCLRE1C* gene point variants/exon deletions. Since then, an increasing number of patients have been reported, some of which were found during newborn screening (Rechavi et al., 2017). Notably, some compound variations [copy number variation (CNV) + single nucleotide variation (SNV)] in the *DCLRE1C* were identified by Sanger sequencing or PCR (Moshous et al., 2001, 2003). However, these traditional methods (Sanger or PCR) can only sequence short DNA fragments. The development of next-generation sequencing (NGS) has accelerated genomics studies. NGS can simultaneously sequence more than 100 genes and has a shorter turnaround time. To date, several studies (Luk et al., 2017; Sundin et al., 2018; Strand et al., 2020) have reported the use of NGS for the detection of SCID patients, and some patients with *DCLRE1C* variants were identified. However, the detection of CNV by NGS remains a challenge because of the issues intrinsic to the technology (such as short read lengths, etc.; Moreno-Cabrera et al., 2020). Thus, some deletions in *DCLRE1C* may be missed by NGS.

Here, we present four cases of SCID caused by *DCLRE1C* variants. All patients were diagnosed with SCID by a physician (two of them were identified during newborn screening). The genetic information of SCID patients carrying the *DCLRE1C* variants was collected from reported studies. The purpose of this study was to expand knowledge related to the genetic information of SCID patients carrying the *DCLRE1C* variant.

## Materials and Methods

### Patients

Four patients were included in the present study. Informed consent was obtained from the parents or guardians of the patients. The study was approved by the Ethics Committee of the Children’s Hospital of Fudan University.

### Whole-Exome Sequencing

At least 2 ml peripheral blood was obtained from the patients and their parents into tubes with EDTA. Genomic DNA was extracted using a TIANGEN DNA Blood Mini Kit. Subsequently, the DNA fragments were enriched using the Agilent SureSelect XT Human All Exon V5 kit. Sequencing was performed on an Illumina HiSeq X10 platform. The average on-target sequencing depth was 120X. Burrows-Wheeler Aligner was used to align the sequencing reads to the reference genome hg19 (Li and Durbin, 2009). The variants were called based on the genome analysis toolkit Best Practices, and a variant call format file was generated. The detailed methods for the data analysis are present in our published study (Yang et al., 2019). For CNV analysis, pipeline for clinical NGS-involved CNV detection (PICNIC) was used to detect CNV from whole-exome sequencing (WES) data. The PICNIC can filter out high-frequency gene deletions/duplications (which occurred in >10% of the internal samples). In addition, we used Database of Genomic Variants, the Database of Chromosomal Imbalance and Phenotype in Humans using Ensemble Resources, and our internal database (Wang et al., 2014) for region-level annotation. More information about CNV detection can be seen in our previous study (Dong et al., 2020).

### Quantitative PCR to Detect DNA

Deletions in the *DCLRE1C* were confirmed using quantitative PCR (qPCR). The primers were designed using Primer Premier 5.0. The details on the primers are shown in Supplementary Table S1. The qPCR mixture (10 μl) contained 5.0 μl SYBR Green mix (2X), 0.2 μl ROX Reference Dye II (50X), 0.5 μmol/L of each primer for the target region and for lactate dehydrogenase A as the reference gene, 20 μg DNA, and ddH_2_O. The following conditions were used for qPCR: an initial 45-s 95°C period followed by 40 cycles of amplification (5 s at 95°C, 30 s at 60°C, and 30 s at 72°C) using the real-time PCR system (Applied Biosystems StepOnePlus). Relative quantification was performed using the 2^-ΔΔCt^ method. The copy numbers that were less than 0.8, between 0.8 and 1.4, and more than 1.4 mean deletion, normal, and duplication, respectively.

### Sanger Sequencing

Sanger sequencing was performed to validate the identified variants. Sequencing was conducted using an automated sequencer (3500XL Genetic Analyzer, Applied Biosystems, United States). The primers were designed using Primer Premier 5.0. The Mutation Surveyor (SoftGenetics®, State College, PA, United States) was used for sequence analysis.

## Results

### Clinical Characteristics

All the clinical features of our patients are listed in Table 1.

**Table 1:** Clinical characteristics of the included four patients.

	Patient 1	Patient 2	Patient 3	Patient 4
Clinical characteristic				
Age	3 months	2 months	3 months	2 months
Gender	Female	Male	Female	Female
Birth weight	NA	3,000 g	2,750 g	3,350 g
Symptoms	Severe pneumonia	Fever, infection	Acute upper respiratory infection, septicemia	Fever, cough
Clinical diagnosis	SCID	SCID	SCID	SCID
Clinical immuno-function test				
CD3^+^% (64–73%)	Decreased	52.06%	54.46%	39.24%
CD4^+^% (29–36%)	NA	25.46%	41.14%	36.16%
CD8^+^% (24–34%)	NA	26.63%	7.97%	2.97%
CD19^+^% (14–21%)	Decreased	0.19%	17.95%	0
CD16^+^CD56% (11–23%)	NA	46.71%	22.59%	56.95%
IgG (3.7–8.3 g/L)	NA	6.1	2.2	7.6
IgA (0.14–0.5 g/L)	NA	2.66	0.08	0.04
IgM (0.33–1.25 g/L)	NA	0.16	0.74	0.04
TREC	Abnormal	NA	NA	Abnormal
Genotype features				
Region of deletion	Exons 1–5	Exons 1–3	Exons 1–3	Exons 1–4
Zygote of deletion	Homozygous	Heterozygous	Heterozygous	Heterozygous
Mutations	/	Exon1: c.92T>C (p.L31P)	Exon5: c.328C>G (p.L110V)	Exon6: c.449_450insT
Zygote of mutation	/	Heterozygous	Heterozygous	Heterozygous
Clinical outcome				
Prognosis	Died	Refused HSCT	Refused HSCT	HSCT
Last follow-up	Died at 4 months old	10 months and 14 days	1 year and 7 months	1 year and 3 months

NA means not available.

Patient 1 (P1) had severe pneumonia at 3 months of age. Her sputum test revealed an infection with *Pneumocystis carinii* and *Candida albicans*. Flow cytometry revealed a decrease in the expression of CD3 and CD19. She died at 4 months of age after unclear treatment at a local hospital. Blood samples were sent to the laboratory for genetic testing.

Patient 2 (P2) developed eczema all over the body without any known cause at 2 months of age. At 4 months of age, the patient was diagnosed with pneumonia due to cough and a recurrent fever. Subsequently, he was diagnosed with *Acinetobacter baumannii* infection and had a recurrent fever, which was identified at a local hospital. He was admitted to our hospital for further treatment at 6 months and 15 days of age. However, the family refused hemopoietic stem cell transplantation at 10 months and 14 days of age.

Patient 3 (P3) presented with fever and vomiting at 4 months of age. She was diagnosed with acute upper respiratory infection, septicemia, and low white blood cell count at a local hospital. She was treated with antibiotics, but the patient frequently had a fever. She visited our hospital for further treatment at 9 months and 3 days of age. However, her parents refused hematopoietic stem cell transplantation, and there was no further follow-up after 1 year and 10 months of age.

Patient 4 (P4) started coughing without a known reason at 2 months of age. Her parents used Chinese herbs, but her symptoms did not improve. At 4 months of age, she had a fever, which increased up to 39.3°C. In addition, an approximately 4-cm mass was found in the left axilla. She was diagnosed with immunodeficiency and tuberculous infection. Her genetic test at the local hospital showed no variation in *RAG1* and *RAG2*. The patient was subsequently referred to our hospital for further treatment at the age of 6 months. She underwent hematopoietic stem cell transplantation at the age of 7 months. Follow-up at 15 months showed normal growth and development.

### Genetic Testing Results

**Figure 1:**
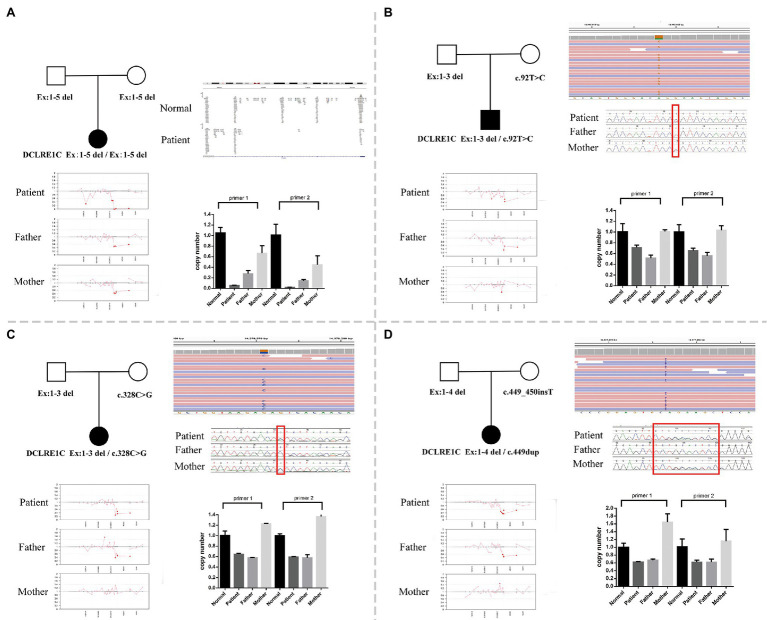
**(A)** A family pedigree of patient 1. Whole-exome sequencing (WES) revealed a homozygous deletion of DNA cross-link repair 1C (*DCLRE1C*) gene from exons 1–5 in this patient, and quantitative PCR (qPCR) verified it. **(B)** A family pedigree of patient 2. WES revealed exons 1–3 deletion and a novel rare variant (c.92T>C, p.L31P). The qPCR verified exons 1–3 deletion, and Sanger sequencing confirmed the variant (c.92T>C, p.L31P). **(C)** A family pedigree of patient 3. WES revealed heterozygous exons 1–3 deletion and a novel rare variant (c.328C>G, p.L110V). The qPCR verified exons 1–3 deletion, and Sanger sequencing confirmed the variant (c.328C>G, p.L110V). **(D)** A family pedigree of patient 4. WES revealed heterozygous exons 1–4 deletion and a novel frameshift variant (c.449_450insT). The qPCR verified exons 1–4 deletion, and Sanger sequencing confirmed the variant (c.449dup, p.His151Alafs*20).

Whole-exome sequencing was performed on peripheral blood sample obtained from the patients and their biological parents. In this study, both the CNV and SNV results were generated. Genetic testing revealed a homozygous deletion of the *DCLRE1C* gene from exons 1–5 in P1 (Figure 1A). P2 had exons 1–3 deletion and a mosaic novel rare variant (c.92T>C, p.L31P; Figure 1B); the short tandem repeat test showed that maternal cells accounted for 31.36% in patient 2. P3 had a compound heterozygous exons 1–3 deletion and a novel rare variant (c.328C>G, p.L110V; Figure 1C). P4 had a heterozygous exons 1–4 deletion and a novel frameshift variant (c.449dup, p.His151Alafs*20; Figure 1D). All variants and deletions of each patient were confirmed by Sanger sequencing and qPCR, respectively (Figure 1). Supplementary Table S2 shows the prediction of pathogenicity for novel variants.

### Genetic Features in Published Studies

Combining Human Gene Mutation Database (HGMD) and PubMed, a total of 26 publications that reported SCID related to *DCLRE1C* until March 2021 were included for analysis. The genetic characteristics of patients from published studies (Moshous et al., 2001, 2003; Li et al., 2002; Kobayashi et al., 2003; Noordzij et al., 2003; Musio et al., 2005; Evans et al., 2006; Darroudi et al., 2007; van der Burg et al., 2007; Lagresle-Peyrou et al., 2008; Alsmadi et al., 2009; Pannicke et al., 2010; Woodbine et al., 2010; Ijspeert et al., 2011; Lee et al., 2011, 2013; Tomashov-Matar et al., 2012; Bajin et al., 2013; Halbrich et al., 2013; Schuetz et al., 2014; Felgentreff et al., 2015; Lobachevsky et al., 2015; Volk et al., 2015; Al-Mousa et al., 2016; Dvorak et al., 2017; Luk et al., 2017; Rechavi et al., 2017; Stray-Pedersen et al., 2017; Tahiat et al., 2017; Al-Herz et al., 2018; Sundin et al., 2018, 2019; Krantz et al., 2019; Wu et al., 2019; Dasouki et al., 2020; Fayez et al., 2020; Firtina et al., 2020; Kalina et al., 2020; Simon et al., 2020; Strand et al., 2020; Vignesh et al., 2020) are listed in Supplementary Table S3. In total, 87 variants were recorded in HGMD as SCID. A combined literature review was performed on studies published on PubMed, and 153 patients were reported with the *DCLRE1C* variants. A total of 116 patients had detailed zygosity information. Of them, 14 patients were compound heterozygous, 10 patients were heterozygous, and 92 patients were homozygous. In total, 37 studies indicated the test methods. Of these studies, 18 studies used PCR, 15 studies (including eight WES) used NGS, two studies used Sanger sequencing, one study used a genome-wide scan, and one study used *multiplex ligation*-*dependent probe amplification*. We observed that the exon deletions were approximately one-third (55/153) of the total variations in SCID patients carrying *DCLRE1C* variants. Among these deletions, more than 50% (33/55) were exons 1–3 deletion; exons 1–3 was recognized as a hotspot region for deletion variation. Moreover, compound variations (CNV + SNV) accounted for approximately 7% (11/153) of variations in all variants.

## Discussion

In this study, four SCID patients with deletions in exons 1–5 were identified. P1 had homozygous exon deletions, while P2, P3, and P4 carried compound heterozygous variants (CNV + SNV). In all the patients, these deletions were inherited from the father, while the point variants were inherited from the mother. We propose the following as a possible mechanism for the observations made in this study. P1 had homozygous exons 1–5 deletion, which is caused by homologous recombination and results in SCID. P2 and P3 carried compound heterozygous variants with exons 1–3 deletion and a missense variant. Exons 1–3 deletion can account for the absence of ARTEMIS protein expression due to homology and should be considered a null allele (Pannicke et al., 2010). These two novel missense variants (p.L31P and p.L110V) are located on exons 1 and 5, respectively. Exons 1 and 2 are important for ARTEMIS endonucleolytic features (Pannicke et al., 2004). Exons 5 and 6 are necessary for maintaining the ARTEMIS structure (Pannicke et al., 2004). Since exons 1–3 deletion is a null allele, p.L31P probably influences the active site of ARTEMIS, and p.L110V likely affects the protein structure, thereby leading to SCID. P4 had a heterozygous exons 1–4 deletion along with a frameshift variant (c.449dup) in exons 6. Felgentreff et al. (2015) verified that a frameshift variant can disrupt domains and almost completely abrogate the ARTEMIS function. We speculated that this frameshift variant probably affects the balance of the ARTEMIS structure and results in SCID.

Our literature review analyzed 26 publications and summarized that exons 1–3 is a hotspot region for deletion. The major mechanism for this hotspot deletion is homologous recombination (Meek et al., 2004). Moreover, Pannicke et al. (2010) found that there was a series of short interspersed nuclear element, long interspersed nuclear element, long terminal repeat, simple repeat, and low complexity repeat sequences in this region that can enhance the recombination events. Although the above-mentioned mechanisms have been identified, more evidence from functional studies are needed.

In addition, our literature review found that compound variations (CNV + SNV) accounted for approximately 7% of the total variants, and deletion in *DCLRE1C* gene was mainly tested by PCR. Currently, NGS is a more powerful tool for obtaining genetic test results than PCR. However, small CNV calling remains challenging due to variable coverage and data analysis pipelines (Teo et al., 2012). WES analysis pipelines still leave a gap of small deletion calling between SNVs/small insertions/deletions and CNVs larger than 1 Mb. In recent years, the development of long-read sequencing, directly sequencing single molecules of DNA in real time, can help for the detection of complex chromosomal rearrangements including deletions, inversions, insertions, and duplications (McGinty et al., 2017). However, long-read sequencing has several limitations, such as high sequencing error rate, high systematic error, and higher cost than NGS (Xiao and Zhou, 2020).

Traditional exome sequencing mainly concentrates on the detection of SNVs. The small deletion may be missed by these methods. Subsequently, several algorithms, including CANOES (Backenroth et al., 2014), XHMM (Fromer et al., 2012), and Co NIFER (Krumm et al., 2012), have been developed for CNV analysis, and recent studies (Yang et al., 2013; Retterer et al., 2015; Ellingford et al., 2018) showed the good performance of CNV analysis through WES. In this study, we used PICNIC (Qin et al., 2018), which demonstrated 100% specificity and sensitivity to pathogenic/likely pathogenic CNVs, for the detection of CNVs. Totally, four cases were diagnosed with SCID, with three compounds heterozygous and one homozygous deletion of the *DCLRE1C* gene, which was detected by WES. Without PICNIC, these cases may have been left undiagnosed if the deletion calling was missed. In addition, qPCR can be further used for deletion validation due to the limitations of NGS. Notably, healthy individuals can also harbor variations in the *DCLRE1C* gene. Thus, healthcare professionals should consider the possibility of deletion when a variant is found in the *DCLRE1C* gene.

Currently, newborns are being screened for SCID in some countries by assessing T-cell receptor excision circles (TREC). Krantz et al. (2019) reported two familial patients diagnosed with SCID who carried the same compound *DCLRE1C* deletions. However, the outcomes for these patients were different: the one who underwent TREC screening and early genetic diagnosis survived with SCID prior to the onset of infections. Although TREC is useful for SCID screening, SCID diagnosis should be confirmed by genetic testing. Although only 5% of SCID patients carry a variant of *DCLRE1C*, these patients (87.5%, 7/8) had 10-year survival rate higher than that of *RAG1/2* SCID patients (64.4%, 9/13) after hematopoietic stem cell transplantation (Hamid et al., 2018). Therefore, the early diagnosis of *DCLRE1C* SCID may provide patients with effective treatment and a better chance of survival.

In conclusion, we reported four SCID cases with five allelic deletions in exons 1–5. When patients are screened for the presence of TREC to diagnose SCID, NGS can be used for further genetic testing. Gross deletions in the *DCLRE1C* should not be ignored when a variant has been identified.

## Data Availability Statement

The datasets for this article are not publicly available due to concerns regarding participant/patient anonymity. Requests to access the datasets should be directed to the corresponding authors.

## Ethics Statement

The studies involving human participants were reviewed and approved by the Ethics Committee of Children’s Hospital of Fudan University. Written informed consent to participate in this study was provided by the participants’ legal guardian/next of kin. Written informed consent was obtained from the minor(s)’ legal guardian/next of kin for the publication of any potentially identifiable images or data included in this article.

## Author Contributions

HW, FX, and WZ conceptualized the study and analyzed the data. YL, BW, BL, GL, PZ, QZ, and JS co-conceptualized the study and interpreted the data. All authors contributed to the drafting of the article and revised it critically for important intellectual content. All authors contributed to the article and approved the submitted version.

## Conflict of Interest

The authors declare that the research was conducted in the absence of any commercial or financial relationships that could be construed as a potential conflict of interest.

## Publisher’s Note

All claims expressed in this article are solely those of the authors and do not necessarily represent those of their affiliated organizations, or those of the publisher, the editors and the reviewers. Any product that may be evaluated in this article, or claim that may be made by its manufacturer, is not guaranteed or endorsed by the publisher.
