# Supplementary material for: High-Frequency Exon Deletion of DNA Cross-Link Repair 1C Accounting for Severe Combined Immunodeficiency May Be Missed by Whole-Exome Sequencing
Source: Front Genet. 2021 Aug 4;12:677748. doi: 10.3389/fgene.2021.677748 (PMC8372405; doi:10.3389/fgene.2021.677748)
Supplement: Supplementary file 1 [file Table_1.DOCX]

Supplementary table 1: Primers for the qPCR

| Primer 1 | Forward | TTACGCTGGCACAAAATCTG |
| --- | --- | --- |
|  | Reverse | ATGTGTGGCGGTATCTTTCC |
| Primer 2 | Forward | GGGTGATGCGTTCATTTTGT |
|  | Reverse | AAGACGACTGTTTCCCTTGG |
| LDHA* | Forward | AAACGGCCAAGACATACAGG |
|  | Reverse | AAGCTGGCAACCACACTTCT |

*normal control
