# Supplementary material for: High-Frequency Exon Deletion of DNA Cross-Link Repair 1C Accounting for Severe Combined Immunodeficiency May Be Missed by Whole-Exome Sequencing
Source: Front Genet. 2021 Aug 4;12:677748. doi: 10.3389/fgene.2021.677748 (PMC8372405; doi:10.3389/fgene.2021.677748)
Supplement: Supplementary file 2 [file Table_2.DOCX]

Supplementary table 2: The prediction of pathogenicity for novel variants.

|  | SIFT_  score | SIFT_  prediction | Polyphen2_score | polyphen2_prediction | MutationTaster_score | MutationTaster_prediction |
| --- | --- | --- | --- | --- | --- | --- |
| c.92T>C | 0 | D | 1 | D | 1 | D |
| c.328C>G | 0.04 | D | 0.939 | D | 1 | D |
| c.449_450insT | / | / | / | / | / | / |

Note: SIFT, “D” meaning deleterious, score less than 0.05; PolyPhen2, “D” meaning probably damaging, 0.957≤ score ≤1; MutationTaster, “D” represents as disease-causing meaning likely deleterious.
