# Supplementary material for: High-Frequency Exon Deletion of DNA Cross-Link Repair 1C Accounting for Severe Combined Immunodeficiency May Be Missed by Whole-Exome Sequencing
Source: Front Genet. 2021 Aug 4;12:677748. doi: 10.3389/fgene.2021.677748 (PMC8372405; doi:10.3389/fgene.2021.677748)
Supplement: Supplementary file 3 [file Table_3.DOCX]

**Supplementary Table 3: Reported SCID patients with DCLRE1C mutations**

| Number of patients | Allele 1 | Allele 2 | Zygosity | Test methods | PMID | Year |
| --- | --- | --- | --- | --- | --- | --- |
| 1 | deletion (exons 1–4) | c.241C>T; p.R81* | Heterozygous | PCR | 11336668 | 2001 |
| 1 | c.241C>T; p.R81* |  | homozygous |  |  |  |
| 1 | deletion (exons 5–6) | c.972+1G>C | Heterozygous |  |  |  |
| 1 | c.241C>T; p.R81* | c.917+1G>A | Heterozygous |  |  |  |
| 4 | c.362+1G>T |  | homozygous |  |  |  |
| 3 | deletion (exons 1–4) |  | homozygous |  |  |  |
| 1 | c.780+1delG |  | homozygous |  |  |  |
| 1 | deletion (exons 5–8) |  | homozygous |  |  |  |
| 21 | c.597C>A; p.Y199* |  | homozygous | PCR | 12055248 | 2002 |
| 1 | deletion (exon 10-12) |  | homozygous | PCR | 12406895 | 2003 |
| 1 | c.353G>T; p.G118V |  | homozygous |  |  |  |
| 2 | c.404G>A; p.G135E |  | homozygous |  |  |  |
| 1 | deletion (exon 3) | c.1167_1168insAG; p.(Asp390Argfs*13) | Heterozygous | PCR | 12592555 | 2003 |
| 2 | deletion (exon 3) |  | homozygous |  |  |  |
| 1 | deletion (exon 3) |  | Heterozygous |  |  |  |
| 3 | deletion (exons 1–3) | c.1290_1306del17; p.(Thr432Serfs*16) | Heterozygous | PCR | 12569164 | 2003 |
| 1 | c.1350_1356delAGATTGT; p.(Asp451Lysfs*11) |  | homozygous |  |  |  |
| 2 | deletion (exons 1–3) |  | Homozygous | PCR | 15770702 | 2005 |
| 1 | deletion (exons 1–4) |  | Homozygous |  |  |  |
| 1 | c.1179delT; p.(Phe393Leufs*9) |  | Homozygous |  |  |  |
| 1 | c.47T>C; p.I16T | c.356C>G; p.S119* | compound heterozygote |  |  |  |
| 1 | c.377G>A; p.G126D | c.207_209delGTT; p.(Leu70del) | compound heterozygous | PCR | 16540517 | 2006 |
| 2 | deletion (exons 1-2) |  | homozygous | PCR | 18034425 | 2007 |
| 1 | c.110A>G; p.D37G |  | homozygous |  |  |  |
| 1 | c.1391_1395delGAATC; p.(Gly464Alafs*18) |  | homozygous |  |  |  |
| 1 | deletion (exons 10–12) |  | NA | NA | 17169382 | 2007 |
| 1 | deletion exon 1 |  | NA |  |  |  |
| 1 | deletion (exons 1–3) | c.19C>T; p.Q7* | Compound heterozygous | PCR | 18223550 | 2008 |
| 2 | deletion (exons 1–3) |  | homozygous |  |  |  |
| 1 | c.457G>A; p.G153R | c.95C>T; p.S32F | Compound heterozygous |  |  |  |
| 6 | deletion (exons 1–3) |  | NA | PCR | 19912631 | 2009 |
| 1 | c.306+1delG | c.761A>T; p.H254L | compound heterozygous | NA | 19022407 | 2009 |
| 1 | deletion (exons 1–3) |  | NA | PCR | 19953608 | 2010 |
| 1 | c.(?_38)_2461?del |  | NA |  |  |  |
| 1 | deletion (exons 1-4) |  | NA |  |  |  |
| 1 | c.(?_38)_3061?del |  | NA |  |  |  |
| 1 | deletion (exons 7-8) |  | NA |  |  |  |
| 1 | c.464+210_678+265 del1139 |  | NA |  |  |  |
| 1 | c.281delC; p.(Ser94Phefs*2) |  | NA |  |  |  |
| 1 | c.1050delA; p.(Val351Leufs*5) |  | NA |  |  |  |
| 1 | c.1140_1146delAGTTCAC; p.(Val381Glufs*19) |  | NA |  |  |  |
| 1 | c.362+1G>T; |  | NA |  |  |  |
| 1 | c.362+5G>C |  | NA |  |  |  |
| 1 | c.82G>C; p.A28P |  | NA |  |  |  |
| 1 | c.353G>T; p.Gly118Val |  | NA |  |  |  |
| 1 | c.494A>T; p.D165V |  | NA |  |  |  |
| 1 | c.682C>A; p.H228N |  | NA |  |  |  |
| 1 | c.1147C>T; p.R383* |  | NA |  |  |  |
| 1 | c.512C>G; p.P171R | c.1299_1306dupAGGATGCT; p.(Cys436*) | homozygous | PCR | 20674517 | 2010 |
| 1* | c.247-1G>T; c.247-306del60bp;c.632G>T |  | Compound heterozygous | PCR | 21184155 | 2011 |
| 1 | c.464+1G>A |  | NA | PCR | 21390052 | 2011 |
| 1 | c.973-1777G>C |  | NA |  |  |  |
| 1 | c.1299_1306dupAGGATGCT; p.(Cys436*) |  | homozygous | PCR | 22527898 | 2012 |
| 2 | deletion (exons 1–3) | c.211A>C; p.T71P | compound heterozygous | MLPA | 24230999 | 2013 |
| 1 | c.265A>G; p.T89A | c.247-3T>C | compound heterozygous | NA | 23701501 | 2013 |
| 1 | c.1464delG; p.(Gln488Hisfs*56) |  | homozygous | Genome wide scan | 23911390 | 2013 |
| 4 | c.241C>T; p.R81* |  | homozygous | NA | 24144642 | 2014 |
| 1 | c.362T>C; p.M121T | deletion (exons 1-3) |  | NGS | 25917813 | 2015 |
| 1 | deletion exon 11 |  | heterozygous | PCR | 26151233 | 2015 |
| 1 | c.571C>T; p.R191* | NA |  |  |  |  |
| 1 | c.716delC; p.(Pro239Leufs*46) |  | homozygous |  |  |  |
| 1 | c.1A>C; p.M1? | c.401C>G; p.T134R |  |  |  |  |
| 1 | c.194C>T; p.T65I | NA |  |  |  |  |
| 1 | c.400A>G; p.T134A | del Ex 1  (not  specified) |  |  |  |  |
| 3 | c.194C>T; p.T65I |  | homozygous | WES | 26476407 | 2015 |
| 2 | c.194C>T; p.T65I | c.1669dupA; p.(Thr557Asnfs*21) | compound heterozygous |  |  |  |
| 1 | c.959C>G; p.S320C |  | homozygous | Target NGS | 26915675 | 2016 |
| 1 | c.194C>T; p.T65I |  | homozygous | WES | 27577878 | 2017 |
| 1 | c.632G>T;p.G211V |  | homozygous |  |  |  |
| 1 | c.368T>C;p.L123S |  | homozygous | WES | 27568080 | 2017 |
| 1 | c.597C>A; p.Tyr199X |  | homozygous | NA | 28436970 | 2017 |
| 2 | deletion (exons 1–3) |  | homozygous | PCR | 28747913 | 2017 |
| 1 | c.272G>T; p.G91V |  | homozygous | WES | 28981982 | 2018 |
| 3 | c.403G>A; p.G135R |  | homozygous | WES | 29051008 | 2018 |
| 1 | c.471dupA; p.(Asp158Argfs*13) |  | homozygous |  |  |  |
| 1 | deletion (exons 1–3) | deletion (exons 1–9); | NA |  |  |  |
| 1 | c.17C>A; p.G6E |  | homozygous |  |  |  |
| 3 | c.1299_1306dup; p.Cys436* |  | homozygous | WES | 29167666 | 2017 |
| 1 | deletion (exons 1–3) |  | homozygous |  |  |  |
| 2 | deletion (exons 1-8) |  | compound heterozygous | Targeted genetic sequencing | 30439467 | 2019 |
| 1 | c.58G>C; p.D20H | c.374A>C; p.Q125P | compound heterozygous | WES | 30630113 | 2019 |
| 1 | c.1147C>T; p.R383X |  | NA | PCR | 31717670 | 2019 |
| 2 | c.((?_-38)_246+?del) |  | NA |  |  |  |
| 1 | T817DEL.-CTTT; F273fsX8 |  | Homozygous | WES | 32135276 | 2020 |
| 1 | c.1A>C; p.M1V | c.401A>G; p.T134R | heterozygous | NGS | 32265901 | 2020 |
| 1 | c.560T>G; p.L187X |  | homozygous | NGS | 32445296 | 2020 |
| 1 | c.716_716delC; P239Lfs |  | homozygous |  |  |  |
| 1 | c.241C >T; p.R74X |  | homozygous | Sanger sequencing | 32517885 | 2020 |
| 1 | deletion (exons 1–3) |  | NA | Sanger sequencing and SNP microarray analyses | 32691468 | 2020 |
| 1 | c. 403G> A; p.G135R |  | NA |  |  |  |
| 1 | c.82C>G; p.Ala28Pro |  | homozygous | gene panel | 32754152 | 2020 |
| 10 | deletion (exons 1–3) |  | Homozygous | NGS | 33628209 | 2021 |
| 1 | c.874dupA; p.M292NfsX33 |  | homozygous |  |  |  |

* This patient has three alleles.
